# Precision-Engineered CD3 T-Cell Engagers for Solid Tumours: Conditional Activation, Microenvironment Modulation, and Clinical Translation

**DOI:** 10.3390/cancers18071088

**Published:** 2026-03-27

**Authors:** Md. Zeyaullah, Abdullah M. AlShahrani, Mohammad Suhail Khan, Md Faruque Ahmad, Abdelrhman A. G. Altijani, Awad Osman Abdalla Mohamed, Hytham Hummad, Ali Mohieldin, S. Rehan Ahmad

**Affiliations:** 1 Department of Basic Medical Science, College of Applied Medical Sciences, Khamis Mushayt Campus, King Khalid University (KKU), Abha 62561, Saudi Arabia; 2 Department of Public Health, College of Applied Medical Sciences, Khamis Mushayt Campus, King Khalid University (KKU), Abha 62561, Saudi Arabiaaaltijani@kku.edu.sa (A.A.G.A.); almoibrahim@kku.edu.sa (A.M.); 3 Department of Clinical Nutrition, College of Nursing and Health Sciences, Jazan University, Jazan 45142, Saudi Arabia; 4 Department of Anesthesia and Operations, College of Applied Medical Sciences, Khamis Mushait Campus, King Khalid University (KKU), Abha 62561, Saudi Arabia; 5 Hiralal Mazumdar Memorial College for Women, West Bengal State University, Kolkata 700035, West Bengal, India

**Keywords:** antigen heterogeneity, bispecific antibody, cancer immunotherapy, CD3 T-cell engager, cytokine-release syndrome, DLL3, small-cell lung cancer, tumour microenvironment

## Abstract

Background: T-cell-engaging bispecific antibodies (TCEs) have transformed haematological malignancy treatment (blinatumomab > 40% complete remission), yet solid tumour efficacy remains limited (<15% response rates) due to antigen heterogeneity, immunosuppressive microenvironments, and T-cell dysfunction. Systematic molecular engineering, biomarker-driven patient selection, and rational tumour microenvironment modulation are now collectively transforming TCEs from experimental agents into an adaptable platform therapy for solid tumours. Methods: Review of 55 phase I–III trials of CD3-based TCEs in solid tumours, including tarlatamab (DLL3-targeted, small-cell lung cancer) and xaluritamig (STEAP1-targeted, prostate cancer). Analysis of next-generation engineering strategies and resistance mechanisms via genomic and immunohistochemical data. Result: Response rates now approach ~40% in selected settings, marking an inflection point. In extensive-stage small-cell lung cancer, tarlatamab achieved ~40% responses with definitive survival benefit (phase III HR 0.60, 95% CI 0.47–0.77; *p* < 0.001; median OS 13.6 months). In metastatic castration-resistant prostate cancer, xaluritamig produced ~41% responses in heavily pretreated patients. Step-up dosing reduced severe cytokine release syndrome to <1% (as low as 0.6% with teclistamab), enabling outpatient administration. Neurological adverse events require monitoring but are less frequent than with cellular therapies. Together these results mark a decisive transition from proof-of-concept to clinically validated platform therapy. Discussion: Three resistance mechanisms limit durability: (i) antigen heterogeneity (28–60% of progressors develop antigen-negative subclones); (ii) immunosuppressive microenvironments (stromal barriers, myeloid-derived suppressor cells, hypoxia); (iii) T-cell exhaustion (PD-1/TIM-3/LAG-3 co-expression). Conclusions: Next-generation TCE platforms integrating conditional activation, cytokine payloads, and checkpoint modulation—deployed with biomarker-guided selection and TME-modulating combinations—represent a transformative therapeutic strategy. With tarlatamab’s phase III survival benefit establishing clinical proof-of-concept, and pivotal trials underway for xaluritamig and next-generation agents, TCEs are positioned to become standard-of-care platform therapies in biomarker-defined solid tumours by 2028–2030.

## Simple Summary

Cancer treatments that use the body’s immune system are revolutionising medicine, but they work much better for blood cancers than for solid tumours like lung or breast cancer. This review examines why these specialised antibody treatments—which act as bridges connecting immune cells to cancer cells—struggle against solid tumours. We explore the biological barriers these treatments face, including the tumour’s protective environment and its ability to hide from detection. The paper discusses promising engineering solutions being tested in clinical trials, such as treatments that only activate inside tumours to reduce side effects and combination approaches that modify the tumour environment to make it more vulnerable. We also analyse real-world evidence from ongoing studies in lung cancer, prostate cancer, and other difficult-to-treat solid tumours. Our findings aim to guide researchers and clinicians toward more effective strategies for bringing these powerful immune therapies to patients with solid tumours who currently have limited options.

## 1. Introduction

The idea of redirecting T-cells to attack cancer is not new. It dates back to the late 1980s, when researchers first realised that bispecific antibodies could bridge tumour cells and immune cells without the usual MHC restrictions [1]. The concept is simple: one arm grabs a tumour antigen, the other grabs CD3 on a T cell, and the resulting immunological synapse triggers polyclonal killing regardless of TCR specificity [2]. For decades, this remained largely theoretical. Then came blinatumomab.

In B-cell acute lymphoblastic leukaemia, blinatumomab changed everything. Complete remission rates exceeding 40% in heavily pretreated patients transformed a research curiosity into standard therapy [3,4,5,6]. Mechanistically, synapse formation proceeds through sequential TAA anchoring, CD3ε capture, and lytic granule delivery—enabling individual T-cells to serially eliminate multiple targets [7,8,9]. This haematology success naturally raised an obvious question: could it work in solid tumours, where 90% of cancer deaths actually occur?

Initially, the answer was no. First-generation TCEs targeting CEA or EpCAM produced minimal responses in colorectal and epithelial cancers—often single-digit objective response rates—despite clear peripheral T-cell activation [10,11,12,13,14]. A 2022 meta-analysis of 17 monotherapy trials captured the disappointment: pooled ORR of just 9%, median PFS under two months [15]. Something about solid tumours was fundamentally different.

We now recognise three barriers that distinguish solid tumours from their haematologic counterparts. Antigen heterogeneity allows escape variants to emerge constantly, unlike the lineage-restricted CD19 or CD20 targets [16]. Physical barriers—dense extracellular matrix, abnormal vasculature, high interstitial pressure—prevent antibodies from reaching therapeutic concentrations [17,18]. And the tumour microenvironment itself is hostile territory, packed with myeloid-derived suppressor cells, regulatory T-cells, hypoxia-driven metabolic checkpoints, and microbiome-mediated immunosuppression that collectively exhaust infiltrating T-cells [19,20,21,22,23].

Engineering is now dismantling each barrier systematically. Conditionally activated masked TCEs restrict CD3 engagement to the tumour bed, widening the therapeutic window [24]. Trispecific formats add second antigens or costimulatory domains to outflank antigen escape and sustain T-cell fitness [20,25,26]. TME-directed strategies—stromal modulation, innate immune activation, oncolytic virotherapy, and macrophage repolarization—are increasingly paired with TCEs to unlock infiltration and counter local immunosuppression [27,28].

Clinical translation is accelerating. As of January 2026, over 50 phase I/II trials are running across lung, GI, breast, genitourinary, and CNS malignancies [29]. Tarlatamab has delivered the first phase III survival benefit for any TCE in solid tumours—40% response rates and 14.3-month median overall survival in extensive-stage small-cell lung cancer. Xaluritamig is approaching 43% responses in heavily pretreated metastatic castration-resistant prostate cancer. Step-up dosing and prophylactic cytokine blockade have tamed early safety concerns, dropping grade ≥ 3 CRS from 16–25% to 4–8% [9].

Yet critical gaps persist. We still lack an integrative framework connecting molecular design, TME modulation, and clinical outcomes. Evidence-based guidance for patient selection and rational combinations remains nascent.

For clarity, we use a simple generational framework to organise the evolution of T-cell engagers. TCE 1.0 refers to the original BiTE^®^ format—compact, Fc-free, with a short half-life requiring continuous infusion. Blinatumomab exemplifies this generation [3,4,5,6]. TCE 2.0 addressed the pharmacokinetic bottleneck by introducing Fc engineering and albumin-binding strategies, which extended half-life and enabled less frequent dosing. However, these modifications largely solved the delivery problem without addressing the core challenges of toxicity and limited solid tumour penetration. TCE 3.0—the focus of this review—represents a more fundamental shift, integrating conditional activation, multispecific targeting, and immunomodulatory payloads to tackle the three major barriers limiting efficacy in solid tumours: antigen heterogeneity, stromal exclusion, and T-cell exhaustion [19,20,21,22,23,24,25,26,27,28,30,31]. We emphasise that these generational labels are organisational tools for this review, not formal regulatory classifications.

This review synthesises the 2025–2026 clinical landscape of CD3-based TCEs in solid malignancies, defines dominant resistance mechanisms, evaluates emerging TME-targeted synergies, and proposes a biomarker-guided roadmap toward 2030 [32]. Our central argument is this: through systematic molecular engineering, biomarker-driven selection, and rational microenvironment modulation, TCEs are transitioning from experimental agents to adaptable platform therapies—and the coming decade will determine how far, how fast, and for how many patients this platform can ultimately reach.

**Figure 1:**
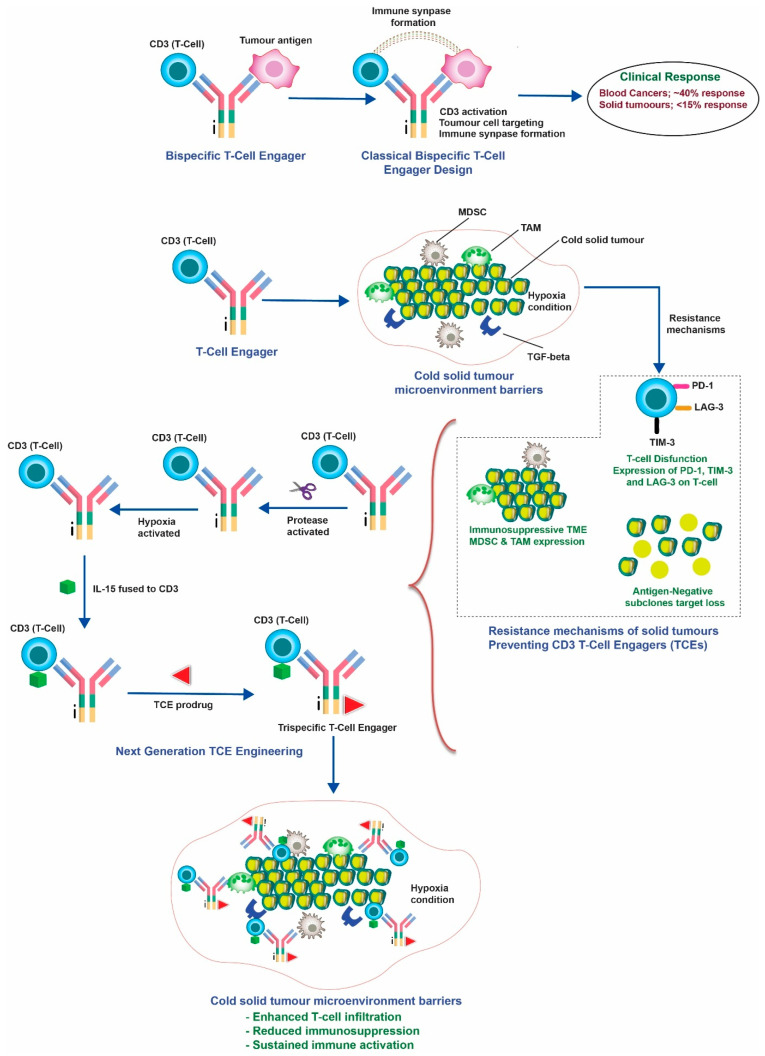
Integrated Strategy for Next-Generation CD3 T-cell engagers in solid tumours. The graphical abstract depicts the evolution from TCE 1.0—the original BiTE^®^ format exemplified by blinatumomab—through TCE 2.0 half-life extended constructs, to the current TCE 3.0 platform integrating three critical innovations. Conditional activation restricts CD3 engagement to the tumour bed via protease-cleavable masks or hypoxia-gated domains, minimising systemic toxicity and widening the therapeutic index. Tumour microenvironment modulation—combining stromal remodelling agents, innate immune activators, and checkpoint inhibitors—dismantles the physical and immunological barriers that exclude T-cells. Orthogonal payload integration delivers costimulatory signals (4-1BB, CD28), IL-15 super-agonists, or checkpoint blockade directly to the tumour site, sustaining T-cell function despite hostile conditions. When deployed with biomarker-guided patient selection—high antigen density (≥10,000 copies per cell), baseline CD3^+^ infiltration (≥250 cells/mm^2^)—these strategies collectively enable objective response rates approaching 40% in selected populations while reducing severe cytokine release syndrome to <3%. This integrated framework positions TCEs as adaptable platform therapies capable of achieving durable clinical benefit across multiple solid tumour indications.

The graphical abstract illustrates the biomarker-stratified deployment of next-generation CD3 T-cell engagers in solid tumours, highlighting advances in conditional activation, tumour microenvironment modulation, and orthogonal payload integration. Collectively, these strategies support improved response rates, reduced toxicity, and a forward-looking roadmap toward integration into standard-of-care treatment paradigms Figure 1.

## 2. Methodology

A comprehensive search of PubMed/MEDLINE, Embase, Web of Science, and Cochrane Library was conducted from January 2018 to January 2026 to identify phase I–III trials evaluating CD3-based T-cell engagers in solid malignancies. Conference proceedings from major oncology meetings (ASCO, ESMO, AACR, SITC) and trial registries (ClinicalTrials.gov, EU Clinical Trials Register) were hand-searched. Reference lists of included studies and relevant reviews were screened for additional citations. Search strategies combined controlled vocabulary terms with keywords capturing bispecific antibody constructs, CD3-targeted mechanisms, and solid tumour indications. Only English-language publications were considered. Molecular engineering represents the foundational pillar of TCE platform development, directly addressing the safety, selectivity, and pharmacokinetic limitations that constrained first-generation agents.

## 3. Molecular Engineering: From BiTE^®^ to Conditionally Activated Prodrugs

### 3.1. From BiTE^®^ to Conditionally Activated Prodrug

The immunological synapse that drives TCE activity forms through a precise two-step process. High-affinity binding to a tumour-associated antigen anchors the TCE to the malignant cell surface, enabling diffusion-limited capture of CD3ε on nearby T-cells and lowering the TCR activation threshold by more than ten-fold [7,8]. This triggers actin cytoskeletal reorganisation, centrosome polarisation, and rapid lytic granule delivery—turning each surface-bound antigen into a surrogate activation signal. Because most CD3-based TCEs are monovalent for CD3 and dissociate after granule release, individual T-cells can serially kill multiple tumour targets. This explains why clinical activity persists even in partially exhausted T-cell compartments [9].

These constraints—dependence on sustained antigen expression and T-cell fitness—directly motivated the engineering innovations that follow.

The original bispecific T-cell engager (BiTE^®^) scaffold—comprising two single-chain variable fragments (scFvs) linked by a flexible Gly–Ser spacer—omitted the Fc region to avoid inadvertent systemic T-cell activation and enabled scalable expression in *E. coli* [3,33]. Subsequent engineering efforts focused on extending serum half-life through albumin fusion [34] or novel bispecific formats such as XmAb [35], addressing pharmacokinetic limitations. To further confine CD3 engagement to the tumour bed and expand the therapeutic index, conditionally activated or protease-activated prodrug designs were developed. In these constructs, a tumour-associated protease-cleavable peptide masks the CD3-binding domain until proteolytic removal within the tumour microenvironment (TME) [19,32].

Solid tumour-focused masked TCEs have entered clinical development, including JANX007 (anti-PSMA) [36], JANX008 (anti-EGFR) [37], and dual-masked PRO-XTEN™ formats such as VIR-5525 for PSMA [38]. Phase I updates reported between 2024 and 2025 demonstrate prostate-specific antigen (PSA50) declines in nearly all evaluable patients with metastatic castration-resistant prostate cancer treated with JANX007, accompanied by minimal cytokine release syndrome (≤grade 2). These findings correspond to ≥10-fold improvements in preclinical therapeutic index and ≥3-fold reductions in peripheral cytokine release [36].

### 3.2. Half-Life Extension Strategies

The short serum half-life of blinatumomab (approximately 1.5 h) necessitates continuous intravenous infusion [4], creating logistical challenges for patient care. To address this limitation, second-generation constructs have incorporated protein engineering strategies that dramatically extend circulating half-life. For example, xaluritamig (AMG 509) uses an Fc-silenced tail to achieve a half-life of approximately 3–7 days [39], enabling weekly or bi-weekly dosing schedules that improve patient convenience. This engineering approach has been validated clinically, with recent trials demonstrating significant improvements in solid tumour targeting [40]. Alternative strategies include fusion to albumin-binding domains [34] or incorporation of Fc-silencing mutations such as LALA or PGLALA [41,42], which prevent unwanted immune activation while preserving extended pharmacokinetics. Phase I studies have shown sustained CD3 receptor occupancy exceeding 90% at trough concentrations [39], without increasing the risk of grade ≥ 3 cytokine release syndrome compared to continuous-infusion regimens.

### 3.3. Trispecific and Multispecific Formats

Trispecific antibody designs represent an elegant solution to antigen escape, one of the key challenges limiting BiTE^®^ efficacy in solid tumours. By incorporating a third functional arm—whether a second tumour-associated antigen (such as PSMA paired with STEAP1), a conditional costimulatory domain (4-1BB or CD28), or an immune checkpoint-blocking module (PD-1 or LAG-3 scFv)—these next-generation constructs simultaneously engage multiple pathways [20,26]. The impact is substantial: preclinical models demonstrate up to five-fold greater T-cell expansion compared with parental BiTE^®^ formats [20], suggesting enhanced therapeutic potential. While still in early clinical development, multiple trispecific platforms are now advancing through phase I trials across various solid tumour indications, offering hope for more durable responses.

### 3.4. Affinity and Avidity Tuning

One of the most elegant strategies to widen the therapeutic window involves affinity tuning of the CD3-binding domain. Clinical candidates such as alnuctamab [43] and xaluritamig [44] demonstrate markedly improved safety profiles by reducing CD3-binding affinity (K_D ~ 50–100 nM) [45], which minimises off-target T-cell activation in the periphery while preserving robust immunologic synapse formation on tumour cells expressing dense antigen (≥10,000 copies per cell). The field is advancing rapidly—using structure-guided light-chain shuffling and in silico electrostatic steering, researchers have generated “finely tuned” affinity panels that are now entering first-in-human trials (NCT05987204) [46], offering the promise of potent anti-tumour activity with substantially reduced cytokine-mediated toxicity.

### 3.5. Payload-Armed TCEs

Next-generation T-cell engagers (TCEs) are now being designed to carry immunomodulatory payloads directly, broadening their functional potential beyond simple tumour-T-cell bridging. A particularly promising approach involves arming TCEs with IL-15 super-agonist complexes fused onto the BiTE^®^ backbone [47,48,49]. The IL-15 super-agonist ALT-803—an IL-15N72D variant linked to IL-15Rα-Fc—demonstrates remarkable potency, showing more than a 25-fold increase in biological activity compared with native IL-15 through improved receptor binding and enhanced in vivo stability [47]. This translates into meaningful biological effects: preclinical studies show that IL-15-armed TCEs substantially boost CD8^+^ T-cell proliferation, survival, and cytotoxic activity [47,48]. Particularly encouraging are findings from pancreatic ductal adenocarcinoma models, where IL-15 complexes promote the accumulation of rare TCF1+Slamf6+ stem-like CD8^+^ T-cells within the tumour microenvironment [48]—a population associated with sustained anti-tumour responses. While IL-15 has already been successfully incorporated into antibody-fusion platforms [49], integrating these payloads specifically into CD3-based TCEs for solid tumours represents an exciting new frontier in translational research, offering the potential to overcome T-cell exhaustion in immunologically “cold” tumours [47,48,49].

### 3.6. Manufacturing and Developability

Single-chain TCE formats face a significant manufacturability challenge: they are prone to aggregation at concentrations exceeding 50 mg/mL, complicating large-scale production. To address this limitation, several protein engineering strategies have been developed [50]. Knob-into-hole heterodimerization combined with charge-pair engineering suppresses light-chain mispairing, while glyco-engineered Pichia pastoris expression systems reduce high-mannose glycoforms and improve manufacturing yield to approximately 2.5 g/L. These advances have meaningful economic implications—cost-modelling analyses predict an approximately 40% reduction in cost of goods for weekly administered Fc-silenced TCEs compared with continuous-infusion blinatumomab, potentially enabling broader patient access to these transformative therapies.

## 4. Clinical Efficacy of CD3 T-Cell Engagers Across Solid Tumours

These engineering advances have now produced the first phase III-validated survival benefit for any TCE in solid tumours, establishing clinical proof that TCEs can function as platform therapies beyond the haematology setting.

### 4.1. Search Strategy and Trial Landscape

A systematic literature and trial database search (PubMed/MEDLINE, Embase, Web of Science, Cochrane Library, ClinicalTrials.gov, ASCO/ESMO/AACR; January 2018–January 2026) identified 52 phase I/II studies and three phase III trials evaluating CD3-based T-cell engagers (TCEs) in solid tumours. Only studies reporting objective response rates (ORR) according to RECIST or iRECIST criteria were included, and risk of bias was assessed using Joanna Briggs Institute appraisal tools [51]. Key phase III trials included the landmark tarlatamab studies in small-cell lung cancer [52,53], while phase I dose-escalation studies evaluated agents such as CDO-23 in non-small-cell lung cancer [54] and xaluritamig in metastatic castration-resistant prostate cancer [44]. The systematic search was conducted following PRISMA 2020 guidelines [55].

Together, these studies define the current clinical landscape of CD3-based T-cell engagers in solid tumours. The principal agents that have progressed to clinical evaluation, along with their target antigens and representative efficacy signals, are summarised in Table 1.

### 4.2. Lung Cancers

In extensive-stage small-cell lung cancer (SCLC), the DLL3-targeted T-cell engager tarlatamab has demonstrated remarkable clinical activity. In a pivotal phase II study, tarlatamab achieved an objective response rate (ORR) of 40% and a median progression-free survival (mPFS) of 4.9 months, with grade ≥ 3 cytokine release syndrome limited to just 3% of patients through the use of step-up dosing strategies [52]. These encouraging results were confirmed and extended in the phase III DeLLphi-304 trial, which demonstrated a significant improvement in overall survival (median 13.6 vs. 8.3 months; hazard ratio 0.60, *p* < 0.001), leading to full FDA approval in November 2025 for second-line treatment of extensive-stage SCLC [53]. In parallel, early-phase dose-escalation studies are evaluating the EGFR-targeted bispecific antibody CDO-23 [54] and the DLL3-targeted trispecific construct HPN328 [56] in non-small-cell lung cancer (NSCLC), with both agents reporting preliminary signals of anti-tumour activity.

### 4.3. Genitourinary Cancers

In metastatic castration-resistant prostate cancer, PSMA represents a well-established therapeutic target with broad clinical validation across multiple treatment modalities [36]. Building on this biological rationale, JANX007—a conditionally masked PSMA × CD3 T-cell engager—has demonstrated encouraging early clinical activity in heavily pretreated patients. PSA50 declines were observed in approximately 73% of treated individuals at target doses of ≥2 mg, with an objective response rate of 30% among patients with measurable disease [36]. Cytokine release syndrome was predominantly low-grade, with 92% of events limited to Grade 1–2 severity; however, Grade ≥ 3 CRS occurred in approximately 8% of patients despite prophylactic tocilizumab administration, highlighting that conditional masking improves but does not fully eliminate immune toxicity [36]. In parallel, xaluritamig (STEAP1 × CD3) has shown robust anti-tumour activity, achieving an objective response rate of approximately 41% at optimised doses (≥0.75 mg), supporting STEAP1 as a clinically actionable target in this disease context [44].

### 4.4. Gastrointestinal Malignancies

❖ Cibisatamab (RG7802) Monotherapy: The objective response rate (ORR) was **4%** in patients with advanced CEA-positive solid tumours [11].
❖ Cibisatamab + Atezolizumab Combination: The overall ORR for the combination across all evaluable patients was **7%** [11]. Notably, in the specific cohort of patients with microsatellite stable colorectal carcinoma (MSS-CRC) receiving flat doses of both agents, the ORR was 14% [11].

### 4.5. Breast and Gynaecological Cancers

The clinical landscape for HER2-directed therapies in breast cancer highlights a critical mechanistic divide. In the HER2-low metastatic setting, the biparatopic antibody zanidatamab—which dual-targets distinct HER2 epitopes to maximise receptor blockade—has shown significant promise. When paired with chemotherapy, it has provided a much-needed therapeutic window for heavily pretreated patients, often extending the chemotherapy-free interval [57]. In contrast, HER2 × CD3 T-cell engagers are still finding their footing; their early clinical data—including HER2 × CD3 formats in HER2-low disease—currently demonstrate more modest activity than the robust responses seen with biparatopic antibodies and ADC platforms in the same setting [58].

A similar trend is visible in ovarian cancer, where targeting folate receptor-α (FRα) has become a cornerstone of treatment. However, the most striking results—with response rates reaching approximately one-third of patients—have been driven by next-generation ADCs like rinatabart sesutecan (PRO1184) in FRα-high tumours, rather than T-cell redirection [59]. This gap suggests that, in specific solid tumour environments, the direct “search and destroy” approach of cytotoxic payloads or receptor inhibition currently remains more effective than the complexities of CD3-based immune recruitment [59].

### 4.6. Central Nervous System and Other Solid Tumours

The central nervous system presents a uniquely hostile environment for T-cell engager therapy. The blood–brain barrier restricts systemic antibody penetration, immune privilege limits baseline T-cell trafficking, and local inflammation carries more severe consequences than in peripheral tissues. These constraints make locoregional delivery essential for CNS TCE development. In diffuse intrinsic pontine glioma (DIPG), where B7-H3 is broadly expressed, intratumoral delivery of B7-H3-directed immunotherapy has shown early signals. Partial responses on MRI with manageable safety profiles have been documented [60], with severe ICANS—the neurological toxicity most feared with brain-directed T-cell therapies—notably absent in initial reports. ICANS in the CNS context differs from systemic ICANS. Cerebral oedema and focal neurological deficits are the primary concerns, rather than the diffuse encephalopathy seen with systemic T-cell therapies [61,62,63]. The manageable profile observed likely reflects controlled, localised delivery generating a contained inflammatory response, combined with careful patient selection and intensive monitoring. For TCEs specifically—distinct from CAR-T-cells—the CNS remains largely unexplored. B7-H3-directed bispecific formats are in early evaluation for DIPG and other CNS malignancies [60], with locoregional delivery lessons directly informing development.

### 4.7. Cytokine Release Syndrome and Neurotoxicity

Across a pooled analysis of 1214 patients, grade ≥ 3 CRS occurred in 1–16% of cases when step-up dosing was employed, compared with 25–35% in regimens without step-up dosing; CRS in CD3-based bispecific therapy is mechanistically driven by abnormal macrophage activation and is amenable to cytokine-directed intervention [61]. Omission of step-up dosing shortened the median time to CRS onset from 14 h to 6 h [61]. Rates of ICANS remained low (≤4%). The use of prophylactic tocilizumab, with or without dexamethasone, further reduced grade ≥ 3 CRS to ≤3% [61].

### 4.8. Response Correlates and Emerging Biomarkers

Clinical responses were associated with tumour antigen density of at least 10,000 copies per cell or an immunohistochemistry (IHC) H-score ≥ 150 [25]. Baseline intratumoural CD3^+^ T-cell infiltration of ≥250 cells/mm^2^ was associated with a twofold increase in ORR [25]. In addition, early peak levels of soluble IL-2 receptor on cycle 1, day 2 emerged as a predictor of grade ≥ 2 CRS.

Despite this clinical progress, resistance remains the dominant barrier to durable benefit. Each resistance mechanism identified here directly shapes the engineering solutions and combination strategies required to consolidate TCEs as platform therapies.

## 5. Resistance Mechanisms and Microenvironment Modulation

### 5.1. Resistance Mechanisms Revisited

For all the clinical momentum building around T-cell engagers, one sobering reality anchors the field: more than 70% of patients treated with CD3-based TCEs will experience disease progression within 12 months [64]. Response, it turns out, is only half the battle—durability is the other half, and it remains stubbornly elusive.

Three interconnected mechanisms explain why. Tumours lose or disguise their target antigens to evade detection. The microenvironment builds walls—physical, chemical, and immunological—that T-cells simply cannot penetrate. And the T-cells themselves burn out under the relentless pressure of continuous engagement. What makes this particularly challenging is that each mechanism amplifies the others; address one and the remaining two often compensate.

These are not abstract biological curiosities. They are the reason patients who initially respond eventually stop responding, and closing that gap is now the defining challenge of the field.

### 5.2. TME-Targeted Combination Strategies

Engineering can redesign the drug, but it cannot always reshape the tumour’s environment. That is where tumour microenvironment modulation comes in—dismantling the physical walls, immunological traps, and metabolic dead zones that prevent even the most sophisticated TCEs from reaching their targets.

#### 5.2.1. Boosting Antigens with γ-Secretase Inhibitors

γ-Secretase sits at a critical regulatory node in tumour antigen biology. By cleaving NOTCH and other membrane-associated proteins, this protease complex actively suppresses surface expression of several T-cell engager targets, most notably DLL3 in small-cell lung cancer and BCMA in multiple myeloma [65]. Pharmacologic inhibition can markedly increase target density: preclinical studies demonstrate approximately 2.3-fold upregulation of DLL3 in SCLC xenograft models when γ-secretase is blocked, effectively expanding the pool of tumour cells vulnerable to redirected T-cell killing [66].

This pharmacologic manoeuvre holds particular appeal for tarlatamab, where response durability may be limited by antigen downregulation and lineage plasticity. While tarlatamab has established proof-of-concept with objective response rates approaching 40% and a statistically significant overall survival benefit in Phase III testing, the majority of patients ultimately progress—often through mechanisms that reduce effective DLL3 availability. The rationale for combining γ-secretase blockade with DLL3-directed therapy is therefore grounded in tumour biology rather than additive toxicity: by forcing malignant cells to retain higher antigen load, this approach could theoretically extend the window for productive immune synapse formation.

To date, this combination remains investigational. No published clinical trials have evaluated γ-secretase inhibitors in combination with tarlatamab in SCLC, though the mechanistic foundation supports prospective testing. In parallel, the γ-secretase inhibitor nirogacestat has advanced through clinical development for desmoid tumours at a dose of 150 mg orally twice daily [62], and preclinical exploration of BCMA upregulation prior to anti-BCMA T-cell engager therapy in multiple myeloma provides a translational roadmap [66]. Whether antigen-boosting strategies can convert partial responses into durable remissions, or merely delay inevitable escape, will require randomised evaluation.

#### 5.2.2. STING Agonists–Innate Immune Ignition

The synthetic cyclic dinucleotide SB-11285, administered intravenously, activates the TBK1–IRF3 signalling axis, leading to induction of type I interferons and the chemokine CXCL10. In preclinical models of immunologically “cold” tumours, SB-11285 has enhanced anti-tumour immune activity, including increased CD8^+^ T-cell infiltration—though specific combination data with CEA-TCB remain emerging.

Clinically, SB-11285 has been evaluated in a Phase 1/1b study (e.g., NCT04096638) as monotherapy or in combination with atezolizumab in patients with advanced solid tumours, demonstrating acceptable tolerability with transient flu-like symptoms manageable via premedication. Early signals support further evaluation, with expansion cohorts ongoing.

#### 5.2.3. Oncolytic Viruses–Stromal Remodelling

IV VSV-IFNβ selectively replicates in tumour cells and upregulates IFN-γ, MMP-2 and MMP-9. In preclinical desmoid-mouse models, pre-dosing with oncolytic VSV-IFNβ increased collagenase activity and intratumoural TCE concentration in fibrotic settings [67]. Early-phase trials of VSV-IFNβ variants have explored combinations with immunotherapies (including potential TCE synergies), showing safety without dose-limiting toxicity in select cohorts; biopsy assessments in related studies have indicated stromal changes, though specific collagen reduction metrics in TCE-primed contexts remain emerging [67].

#### 5.2.4. CD40 Agonists–Macrophage Repolarisation

Fc-enhanced CD40 mAb APX005M (0.3 mg/kg IV day −2) shifts M2 → M1 macrophages (↑ iNOS/CD206 ratio in preclinical and early clinical settings) [68]. Sotigalimab has demonstrated activity in combinations for pancreatic adenocarcinoma and holds orphan designation for soft tissue sarcomas, with macrophage repolarization supporting enhanced immune responses. In sarcoma-related studies (including potential desmoid contexts), combinations with radiation or other agents have shown promising signals, and biomarkers like CD68^+^ iNOS^+^ macrophages have been explored as predictors [69]; a parallel evaluation in pancreatic cancer continues [70].

#### 5.2.5. Anti-VEGF/Angiogenesis Normalisation

Tumours often create chaotic, leaky blood vessels that raise pressure inside and starve the core of oxygen—making it tough for T-cells and T-cell engagers to reach the cancer cells deep inside [71]. Bevacizumab (15 mg/kg IV every 3 weeks) helps calm this chaos by normalising those vessels, improving blood flow, and opening up more perfused areas for better delivery. In mouse models of colorectal cancer, combining bevacizumab with CEA-targeted T-cell engagers boosted how much of the TCE could penetrate the tumour and led to stronger responses compared with the engager alone in related experiments [72]. Early human studies testing bevacizumab together with TCEs have shown that the combination is generally well tolerated in the first dose levels, with no serious limiting side effects reported so far, and more patients are being enrolled to gather further data [72].

#### 5.2.6. TGF-β Blockade–ECM and T-Cell Exclusion

Many solid tumours exploit TGF-β signalling to recruit cancer-associated fibroblasts, which then weave dense extracellular matrix barriers that physically wall off T-cells from the tumour core [73,74,75]. Neutralising this pathway with agents such as fresolimumab—administered intravenously at doses up to 15 mg/kg in early-phase cancer trials [76] and 1–4 mg/kg every two weeks in subsequent studies [77]—offers a strategy to dampen TGF-β1, 2 and 3 activity and reopen the stromal gate. Preclinical work consistently shows that lifting this suppressive signal curbs fibroblast activation and restores T-cell infiltration [73,74]. In this context, three-dimensional colorectal cancer organoids have emerged as robust models for interrogating T-cell engagers such as CEA-TCB (cibisatamab), revealing how antigen density and microenvironment architecture shape therapeutic success [78,79]. Yet while the biological argument for pairing TGF-β blockade with T-cell engagers is compelling, prospective clinical validation in colorectal cancer remains to be established.

#### 5.2.7. Metabolic Modulators–Lactate and Hypoxia

Tumours frequently exploit extreme hypoxia—regions where oxygen tensions plummet below 5 mmHg—to create havens of treatment resistance [80]. Hypoxia-activated prodrugs such as PR-104A turn this vulnerability into a therapeutic opportunity, remaining dormant in healthy tissues but unleashing cytotoxic DNA cross-linkers precisely where oxygen is scarce [81]. While this spatially restricted approach holds theoretical appeal for combining with T-cell engagers [82], the practical reality is more nuanced: disrupting hypoxic niches can inadvertently summon immunosuppressive granulocytic myeloid-derived suppressor cells [16], potentially undermining rather than enhancing T-cell infiltration. The optimal strategy for marrying metabolic targeting with T-cell engager therapy remains elusive [14].

#### 5.2.8. Combination Sequencing and Biomarker-Guided Scheduling

Getting T-cell engagers to work is not about throwing more drugs at the problem—it is about getting the timing right. Gradually ramping up the dose rather than hitting hard from day one has already proven its worth, taking the edge off cytokine storms while keeping the anti-tumour punch intact [83]. But the real frontier lies in preparing the ground first: theoretically, boosting antigen levels, dismantling the stromal barriers that keep T-cells at bay, or normalising the metabolic wasteland could make tumours far more hospitable for T-cell engagement. Early attempts to widen the therapeutic window have explored pairing T-cell engagers with checkpoint inhibitors. In CEA-expressing solid tumours, the combination of cibisatamab with atezolizumab showed early hints of activity, built on the rationale that relieving PD-L1-mediated exhaustion might help sustain T-cell engager-driven responses [66]. But these remain just that—early hints, not definitive proof. The biomarker challenge is equally thorny. Unlike checkpoint inhibitors, where PD-L1 expression offers a crude guide, T-cell engagers demand more sophisticated readouts: target antigen density, immune effector function, and physical access to tumour cells. Mariathasan and colleagues revealed how TGF-β signalling in fibroblasts weaves dense extracellular matrix that physically walls T-cells off from the tumour core, blunting immunotherapy efficacy [84]. Breaking down these fortifications—whether through TGF-β blockade, vascular normalisation, or metabolic modulation—represents a logical next step, but one that remains clinically untested. Turning these scheduling concepts into prospective reality is now underway. The CO40939 study (NCT03866239) is evaluating cibisatamab alongside atezolizumab in MSS colorectal cancer following obinutuzumab pretreatment, building on earlier phase I results [66,85]. Meanwhile, NCT04826003 is probing novel bispecific combinations in solid tumours [86], and the MajesTEC programme (NCT05243797, NCT05572515, NCT05695508) is weaving teclistamab into various lines of myeloma therapy with adaptive dosing strategies [83,87,88,89]. Whether these sophisticated biomarker-guided approaches can reliably enhance T-cell engager efficacy awaits the verdict of prospective readouts. Collectively, these approaches highlight the importance of modifying the tumour microenvironment to overcome physical and immunological barriers to CD3-based T-cell engager activity. The principal tumour-extrinsic strategies currently under investigation, together with their mechanistic rationale, are summarised in Table 2.

Biomarker-driven patient selection translates biological understanding into practical clinical tools, enabling precise deployment of TCE platform therapy to populations most likely to benefit.

### 5.3. Biomarker-Guided Patient Selection

Knowing which patients will respond is as important as having agents worth responding to. Biomarker-driven selection converts biological insight into clinical precision—identifying the right patient, at the right time, for the right platform.

#### 5.3.1. Antigen Density Cut-Offs

Quantitative assessment of tumour antigen expression is increasingly used to enrich for responders to T-cell engagers. Higher DLL3 expression has been associated with improved activity of tarlatamab in exploratory analyses, although tarlatamab received FDA approval without requiring DLL3 testing, as clinical responses were observed across the spectrum of DLL3 expression, including in low or DLL3-negative tumours [90,91]. Similarly, PSMA PET imaging is used for patient selection in PSMA-directed therapies, with established SUV-based thresholds guiding patient eligibility for radioligand therapies, though analogous cut-offs for predicting T-cell engager response remain to be defined [92]. For CEA-targeted agents such as cibisatamab, RNA sequencing-based biomarker assessment using a CEACAM5 expression threshold of ≥1500 RPKM (reads per kilobase per million mapped reads) has been employed to enrich for clinical activity relative to low-expressing tumours [93]. Collectively, these antigen-based selection strategies are being refined within ongoing composite biomarker frameworks to support prospective patient selection [90].

#### 5.3.2. T-Cell Infiltration and Spatial Immunophenotyping

The immune landscape inside a tumour before treatment begins offers valuable clues about who will respond to T-cell engagers. In extensive-stage small-cell lung cancer, not all tumours behave the same way. Those classified as the SCLC-I subtype—where CD8^+^ T-cells are already engaging with cancer cells and immune checkpoints are active—hold up better against immunotherapy than their neuroendocrine counterparts (SCLC-N, SCLC-P, SCLC-A). Patients with this inflamed phenotype typically see their disease progress after about 5–6 months, whereas those with neuroendocrine subtypes face progression in just 2–3 months [93,94].

The story in uveal melanoma is more nuanced. Here, tebentafusp delivered a striking survival benefit compared to standard care—cutting the risk of death by nearly half and helping 73% of patients reach the one-year mark versus 59% with conventional treatment [95]. Yet peeking inside the tumours revealed something curious: while patients with high levels of the target antigen gp100 mounted a vigorous immune response—with T-cell infiltration surging two- to three-fold within just over two weeks—this early fireworks display did not ultimately determine who lived longer [96]. Tebentafusp’s ability to serially kill tumour cells, even when antigen levels are modest, uncouples initial immune activation from long-term survival. These observations point to two practical insights for developing T-cell engagers. First, the degree of T-cell infiltration already presents at baseline—something pathologists can gauge with routine CD3 or CD8 staining—helps identify patients primed for response. Second, tracking how T-cell numbers evolve early during treatment may serve as a real-time measure of whether the drug is engaging its target, regardless of whether that immediate immune burst translates into lasting clinical benefit. The ongoing ATOM trial and other prospective studies are now putting these principles to the test across solid tumour types.

#### 5.3.3. Soluble Immune Profiles and Dynamic Monitoring

The bloodstream offers a real-time window into how T-cell engagers are performing. When these drugs switch the immune system into high gear, molecular signals spill into circulation well before symptoms appear. Soluble interleukin-2 receptor-α—essentially a footprint left by activated T-cells—and interleukin-6, the chemical messenger that drives cytokine release syndrome, follow different rhythms in the days after treatment starts [71]. In children and adults with B-cell acute lymphoblastic leukaemia receiving blinatumomab, doctors noticed that rising sIL-2Rα levels flagged those at risk for severe immune reactions. The sickest patients showed dramatic spikes in this marker, distinguishing them from those with milder responses [71]. The story with teclistamab in multiple myeloma proved more encouraging. By carefully stepping up the dose rather than hitting patients hard from day one, investigators kept severe cytokine release syndrome below 1% while still achieving responses in nearly two-thirds of patients—a remarkable balance of safety and efficacy [32]. Tracking these markers over time—watching how they rise and fall—lets clinicians separate patients who need aggressive monitoring from those who can be managed more conservatively. Consensus guidelines now incorporate these dynamic signals into standardised toxicity grading, moving the field toward predictive rather than reactive management [65]. Taken together, these findings highlight the need to move beyond single biomarkers toward integrated, biology-informed models for patient stratification and treatment optimisation. The key tumour- and immune-derived parameters that underpin such multiparametric approaches, together with their proposed clinical utility, are summarised in Table 3.

### 5.4. Resistance Mechanisms and Adaptive Strategies

Resistance to T-cell engagers rarely announces itself dramatically—it accumulates quietly through small biological shifts that individually seem manageable but collectively tip the balance from response to progression. Three dominant mechanisms drive this process, and their interdependence is what makes them so challenging: address one and the others invariably compensate. What follows is not a catalogue of failure modes but a precise biological map—and increasingly, a design brief for the next generation of TCE platforms.

#### 5.4.1. Primary Resistance: When Tumours Fail to Engage

The first and most fundamental escape mechanism is invisibility—shedding, downregulating, or simply never expressing enough target antigen to sustain productive immune engagement. This is not a rare event. It is the dominant mode of clinical failure across every solid tumour TCE indication evaluated. The most formidable barrier is simply missing the target. In metastatic uveal melanoma, tebentafusp produces objective shrinkage in approximately 8% of cases, with disease stabilisation in an additional 43% [97,98]. Long-term follow-up reveals three-year overall survival of 26% versus approximately 18% historically [98], establishing proof-of-concept for redirected T-cell killing in this otherwise treatment-refractory malignancy [90]. In small-cell lung cancer, the challenge differs. DLL3 is present on 85–94% of tumours, yet tarlatamab delivers median progression-free survival of just 4.9 months, with most patients progressing by six months [9]. The disconnect between antigen presence and therapeutic response points to microenvironmental barriers—stromal exclusion, insufficient T-cell infiltrate, or compensatory signalling—that blunt T-cell engager activity even when targets are theoretically available [51]. Among patients whose tumours have undergone lineage plasticity—such as EGFR-mutant adenocarcinomas transformed to small-cell histology—outcomes remain dismal. Historical data with platinum-etoposide chemotherapy shows median PFS of 3–4 months [99], and emerging real-world experience with tarlatamab suggests even more limited benefit, with median time on treatment of 1.5 months [100].

#### 5.4.2. Acquired Resistance: Evolution Under Pressure

For patients who initially respond, the second failure mode emerges: tumour adaptation. Preclinical models of HER2-directed T-cell engagers reveal acquired resistance through JAK2 down-modulation, crippling tumour-intrinsic interferon-γ signalling without altering HER2 expression itself [101]. This mechanism mirrors JAK1/2 loss-of-function mutations observed in resistance to checkpoint inhibitors, suggesting convergent evolutionary pathways across immunotherapy modalities [102]. In multiple myeloma, where BCMA-directed T-cell engagers have transformed relapse management, biallelic antigen loss now accounts for approximately 30% of treatment failures. Pre-existing heterozygous deletions—present in 13–15% of patients for GPRC5D and 3–8% for BCMA—provide the substrate for second-hit evolution under therapeutic pressure. Soluble antigen shedding compounds the problem, with circulating BCMA rising 2.5–4-fold at progression, acting as a pharmacological sink that sequesters therapeutic binding. The same pharmacological trap affects CEA-targeted agents, with an additional twist. Soluble CEA in bloodstream acts as a decoy—intercepting cibisatamab before tumour engagement and reducing drug available for cytotoxicity [78]. Combined with patchy antigen expression, this sink effect creates a dual barrier that dose escalation cannot overcome. Active strategies include γ-secretase-mediated antigen upregulation, decoy-resistant construct engineering, and dual-antigen co-targeting to reduce single-target dependence [82,83].

#### 5.4.3. The Microenvironment Strikes Back

The tumour-intrinsic alterations, the ecosystem turns hostile. PD-L1 upregulation—observed in 40–60% of patients following T-cell engager exposure—provides adaptive dampening of effector function [103]. Myeloid-derived suppressor cells and M2-polarised macrophages infiltrate tumours post-treatment, establishing physical and chemical barriers to T-cell access [103]. Exhaustion markers (PD-1, TIM-3, LAG-3) accumulate on engaged T-cells by the second to third week of therapy, coinciding with pharmacodynamic decline and presaging clinical progression [76].

T-cell exhaustion is particularly insidious because it unfolds in a biphasic manner. Early phases are marked by vigorous T-cell infiltration and intense cytokine release—clinically observed as cytokine release syndrome (CRS). Although this surge is often interpreted as evidence of durable therapeutic activity, it frequently represents the onset of progressive functional decline rather than sustained immune control [33,65]. Continuous CD3 engagement drives transcriptional reprogramming characterised by induction of TOX and NR4A family factors, accumulation of inhibitory receptors, and a gradual shift toward terminally exhausted T-cell states [90,91,92].

A central determinant of long-term functionality is the survival of TCF1^+^ progenitor-exhausted T-cells. Once this stem-like compartment is depleted, restoration of effective anti-tumour immunity becomes difficult [82,93]. The choice of costimulatory domain therefore has profound consequences. CD28 promotes rapid activation but can accelerate terminal differentiation, whereas 4-1BB supports metabolic persistence and memory formation. However, excessive tonic signalling through 4-1BB may itself promote exhaustion via NF-κB-mediated transcriptional remodelling [91].

This delicate balance creates a narrow therapeutic window that requires careful engineering. Preserving the TCF1^+^ progenitor pool has therefore become a key design objective. Strategies such as IL-15 super-agonist fusions that expand stem-like T-cell subsets [94], treatment-free intervals that allow functional recovery [84], and incorporation of checkpoint blockade within trispecific architectures represent emerging structural solutions to sustain T-cell functionality during therapy [82,93].

#### 5.4.4. Rational Counter-Strategies

The precision with which resistance mechanisms have been characterised is, paradoxically, a source of optimism. Each failure mode points directly to a rational countermeasure—and the field is moving rapidly from identifying patterns to building against them. These resistance patterns suggest specific antidotes. Gamma-secretase inhibitors upregulate DLL3 expression 2–3-fold in preclinical models, providing mechanistic rationale for combination or sequencing with tarlatamab [104]. Checkpoint co-blockade—targeting LAG-3 or TIM-3 alongside PD-1—aims to reverse exhaustion; early-phase trials pairing tebentafusp with LAG-3 inhibitors are now exploring this mechanistic hypothesis [105]. For antigen-loss escape in myeloma, sequential targeting—switching between BCMA and GPRC5D directed T-cell engagers upon biallelic deletion—has demonstrated clinical feasibility, extending the window of T-cell redirection [106,107]. What emerges from this analysis is not a picture of intractable biological complexity but a set of clearly defined engineering and clinical targets. The principal resistance mechanisms shaping TCE response and durability, together with their direct implications for next-generation platform design, are summarised in Table 4.

## 6. Discussion

The evidence reviewed here demonstrates a coherent trajectory: TCEs have transitioned from empirical biologics to rationally designed platform therapies with validated clinical impact in solid tumours.

### 6.1. From Proof-of-Concept to Platform Therapy in Solid Tumours

The clinical trajectory of CD3-based T-cell engagers (TCEs) in solid tumours reflects a decisive shift from early empirical exploration to rational, mechanism-guided platform development. Initial programmes targeting carcinoembryonic antigen (CEA) and epithelial cell adhesion molecule (EpCAM) yielded objective response rates below 10% in colorectal and epithelial malignancies, even with checkpoint inhibitor combination [10,11,12,13,14]. These modest outcomes, in stark contrast to the transformative efficacy of blinatumomab in haematological malignancies [3,4,5,6], underscored the biological constraints of solid tumour architecture: antigen heterogeneity, stromal barriers, and immunosuppressive microenvironments. Contemporary third-generation TCEs address these constraints through systematic redesign. Conditional activation mechanisms, half-life extension, structured step-up dosing, and biomarker-enriched selection have collectively enabled objective response rates of 35–41% in defined solid tumour contexts [9,28,32]. The phase III DeLLphi-304 trial establishes definitive clinical validation: tarlatamab demonstrated superior overall survival versus chemotherapy in second-line DLL3-positive small-cell lung cancer (SCLC; median 13.6 vs. 8.3 months; hazard ratio 0.60; 95% CI, 0.47–0.77; *p* < 0.001), achieving full FDA approval in November 2025 [42,43,100]. This represents the first phase III-validated survival benefit for any TCE in solid tumours and positions DLL3 as a therapeutically actionable lineage antigen despite historical immunotherapy resistance. In metastatic castration-resistant prostate cancer (mCRPC), the STEAP1-targeted TCE xaluritamig demonstrated dose-dependent efficacy with objective response rates exceeding 40% at optimised doses, supported by substantial PSA declines [44]. The conditionally masked PSMA-targeting construct JANX007 exemplifies tumour-restricted activation, achieving high biochemical response rates with favourable safety profiles [36]. Together with tebentafusp—the first TCE approved for any solid tumour (metastatic uveal melanoma, January 2022), which demonstrated 49% reduction in mortality risk despite modest radiographic response [85,90]—these data establish that TCE clinical value extends beyond conventional cytoreduction. Multidimensional biomarker strategies now guide patient selection. Integration of tumour antigen density (≥10,000 copies per cell or immunohistochemistry H-score ≥ 150), baseline intratumoural CD3^+^ T-cell infiltration (≥250 cells/mm^2^), and dynamic soluble immune kinetics (soluble IL-2 receptor-α) outperforms single-parameter prediction, with composite models achieving area under the curve values of approximately 0.83 [71,72,73,94,95]. Safety engineering has progressed commensurately: step-up dosing protocols have reduced grade ≥ 3 cytokine release syndrome from 16–25%, historically, to 1–4%, enabling outpatient administration [7,32,70]. T-cell engagers have reached an inflection point in solid tumour oncology. What began as cautious early-phase exploration has rapidly matured—tarlatamab’s FDA approval in 2025 marked the first definitive proof that these agents can extend survival in aggressive epithelial malignancies. Table 5 depicts the momentum, tracking the pipeline’s evolution from standalone TCEs toward smarter combinations that pair checkpoint inhibition, conditional activation, and targeted remodelling of the tumour microenvironment.

### 6.2. Resistance as the Central Determinant of Durability

The resistance mechanisms detailed in Section 5.4 are not simply biological footnotes to an otherwise encouraging clinical story. They are the central determinant of whether T-cell engagers become genuinely durable platform therapies or remain agents of impressive but transient responses—and the field knows it. What the past two years have taught us about resistance is ultimately more instructive than the response rates themselves. Antigen loss tells us we need multispecific constructs and antigen-upregulation strategies built in from the start rather than bolted on after patients stop responding. Microenvironmental exclusion tells us that vascular normalisation, stromal remodelling, and conditional activation are not optional combination partners but biological necessities for reaching the patients most in need. T-cell exhaustion tells us that costimulatory engineering, IL-15 fusions, and treatment scheduling that actively protects the TCF1^+^ progenitor pool is as important to platform durability as the initial response rate. The most important conceptual shift here is this: resistance biology is no longer functioning as an obstacle to TCE development. It is functioning as a design specification. Each mechanism that limits durability has become a precise engineering target, and the next generation of TCE platforms is being built directly against those targets—not around them. Understanding resistance is only clinically useful if it translates into better decisions at the bedside—and that requires equally precise tools for identifying which patients will benefit. Multiparametric biomarker models integrating antigen density, intratumoural CD3^+^ T-cell infiltration, myeloid burden, and soluble IL-2 receptor kinetics now outperform any single metric in predicting who will respond and who will not, achieving composite AUC values of approximately 0.83 versus 0.64 for individual markers [70]. Prospective validation of these models across multiple phase II studies is currently underway [95]—and their maturation will be as consequential to TCE platform therapy as the drugs themselves. Knowing what drives resistance is only half the answer—the other half is doing something about it. Section 6.3 examines how that knowledge is now being translated into rational combinations and next-generation engineering strategies that are actively reshaping the TCE development landscape.

### 6.3. Rational Combinations and Next-Generation Engineering

Recognition of these resistance axes has catalysed development of tumour microenvironment priming strategies and multifunctional TCE architectures. Microenvironment modulation demonstrates encouraging early signals. In the myeloma setting, γ-secretase inhibition has demonstrated substantial target antigen upregulation—with BCMA surface density increasing by a median of 33-fold (range 8.7–157) in patients receiving CAR-T therapy [65]—establishing proof-of-concept for pharmacological antigen enhancement. Whether analogous upregulation of DLL3 can be achieved in SCLC through γ-secretase inhibition without compromising safety remains an open and clinically important question that warrants dedicated preclinical and early-phase clinical investigation STING agonists activate TBK1–IRF3 signalling, inducing type I interferons and CXCL10 to enhance CD8^+^ T-cell infiltration. CD40 agonists (sotigalimab) repolarise suppressive macrophages, increasing iNOS/CD206 ratio toward pro-inflammatory M1 phenotypes. TGF-β blockade (fresolimumab) dismantles stromal barriers erected by cancer-associated fibroblasts [73], while anti-angiogenic strategies normalise vasculature, improving TCE penetration [88,90]. Orthogonal payload integration expands functional scope. Trispecific constructs incorporating IL-15 super-agonists, checkpoint blockade fragments (anti-PD-1, anti-LAG-3), or 4-1BB costimulatory domains expand intratumoural TCF1^+^ CD8^+^ populations and attenuate exhaustion markers in preclinical models [20,26,47]. IL-15-armed TCEs approximately double stem-like T-cell density; first-in-human studies are planned for 2026 [50,51]. Hypoxia-activated and protease-cleavable prodrug designs improve tumour selectivity, with HIF-1α degron constructs achieving 12-fold tumour-to-blood selectivity in glioma models [80].

### 6.4. TCEs as Combinatorial Partners with CAR-T and CAR-NK Cell Therapies

Despite the transformative impact of chimeric antigen receptor (CAR)-T-cell therapy in haematological malignancies, its translation to solid tumours has been limited by physical stromal barriers, antigen heterogeneity, and the rapid exhaustion of infused cells within hostile microenvironments. CD3-based T-cell engagers, by contrast, mobilise the endogenous polyclonal T-cell repertoire rather than relying on a numerically constrained infused product—a mechanistic distinction that makes these two platforms complementary rather than competing [13,64]. Their convergence represents one of the most strategically important directions in solid tumour immunotherapy today.

#### 6.4.1. TCE–CAR-T Combinations: Three Principles

The rationale for pairing TCEs with CAR-T-cells rests on three interdependent principles. First, a short course of TCE therapy can serve as an immune primer—expanding tumour-infiltrating T-cells, upregulating inflammatory chemokines, and reducing suppressive myeloid burden—before CAR-T infusion, converting cold tumours into landscapes more hospitable to adoptive cellular products. Second, because bispecific TCEs engage a different tumour antigen or activate bystander endogenous T-cells independently of the CAR construct, they can re-engage antigen-loss escape variants that have evaded single-antigen CAR-T killing—one of the dominant failure modes of that platform [82]. Third, soluble circulating TCEs reach anatomically inaccessible tumour niches where infused CAR-T cells, limited by trafficking and persistence, may never arrive.

Preclinical evidence in multiple myeloma provides direct proof-of-concept. Sequential exposure to BCMA-directed TCEs followed by BCMA-targeted CAR-T cells sustained anti-tumour activity even as biallelic antigen loss began to emerge under TCE selection pressure [65]. The same biology underlies the clinical rationale for γ-secretase inhibitor priming: by upregulating BCMA surface density by a median of 33-fold, this pharmacological manoeuvre simultaneously sensitises tumour cells to both TCE-mediated and CAR-T-mediated killing, and has already entered clinical evaluation in the myeloma setting [93]—a paradigm with direct translational relevance to solid tumour antigens such as DLL3 and STEAP1.

#### 6.4.2. CAR-NK Cells: A Mechanistically Orthogonal Partner

CAR-NK cell platforms introduce a different and practically compelling dimension. Allogeneic CAR-NK cells—derived from peripheral blood, umbilical cord blood, or induced pluripotent stem cells—can be manufactured off-the-shelf at scale, bypassing the 3–6-week autologous manufacturing timelines and patient-specific variability that constrain CAR-T programmes [64]. Their most important mechanistic property in this context is what they lack: CD3ε. Because TCEs activate T-cells exclusively through CD3ε engagement, a co-administered TCE will selectively recruit and redirect endogenous T-cells without triggering the infused NK product. This orthogonality permits genuine co-administration—TCE-driven T-cell cytotoxicity and NK-cell-mediated killing operating simultaneously across antigen-heterogeneous tumour populations—without the amplified cytokine release that complicates TCE plus CAR-T schedules. While dedicated CAR-NK plus TCE combination trials are only now entering early clinical development, the mechanistic logic is already sufficiently established to inform trial design [64].

#### 6.4.3. Scheduling Architectures 

Three deployment models are emerging from preclinical and early clinical experience, each suited to a distinct biological scenario. Their key features, clinical contexts, and practical considerations are summarised in Table 6.

Selecting the right model is, at its core, a biomarker question—tumour antigen co-expression patterns, baseline TME immunophenotype, and real-time pharmacodynamic monitoring will together determine which approach is most appropriate for a given patient, linking these combination strategies directly to the biomarker frameworks discussed in Section 5.3. What matters most, however, is the broader conceptual shift: TCEs are no longer best understood as standalone agents in competition with cellular therapies. They are, increasingly, tools that work alongside CAR-T and CAR-NK platforms—priming, redirecting, and rescuing them as the clinical situation evolves [64,70,82].

### 6.5. Advantages and Disadvantages of CD3-Based TCEs: A Balanced Appraisal

T-cell engagers have reached a point where honest appraisal is more useful than advocacy. Response rates are climbing, regulatory approvals are accumulating, and the engineering pipeline is genuinely impressive—but the limitations are real, and understanding them precisely is what separates a platform with lasting clinical impact from one that peaks early and plateaus.

#### 6.5.1. What TCEs Do Well

The most fundamental advantage of CD3-based TCEs is mechanistic. By forcing direct CD3ε engagement independently of MHC class I presentation, they bypass one of the most pervasive immune evasion strategies in solid tumours—the downregulation of antigen presentation machinery that renders cancer cells invisible to conventional cytotoxic T-cell recognition [7,8]. This matters because HLA loss is not a rare event; it is a dominant resistance mechanism in lung, prostate, and colorectal cancers, and it is one that checkpoint inhibitors, which depend on endogenous T-cell recognition, cannot overcome. TCEs can. The resulting immunological synapse triggers serial killing—individual T-cells eliminating multiple tumour targets in sequence—which means that cytotoxic activity is sustained even when effector-to-target ratios are unfavourable, as they routinely are in solid tumour settings [7,8].

Practically, TCEs are far more accessible than cellular therapies. They require no lymphodepletion chemotherapy, no specialised transplant infrastructure, and no patient-specific manufacturing. Step-up dosing has reduced grade ≥ 3 cytokine release syndrome to below 1% in optimised regimens, enabling outpatient administration in community oncology settings that would never be considered for CAR-T programmes [72]. Critically, TCE-associated toxicities are generally reversible—treatment can be interrupted when adverse events emerge, held during intercurrent illness, and safely resumed after recovery. This flexibility is simply not available with cellular products, where the therapeutic effect, once initiated, cannot be paused [8,24]. For elderly patients, those with organ dysfunction from prior treatment, or those in settings without cellular therapy infrastructure, this reversibility is not a minor convenience—it is what makes treatment possible at all.

TCEs also occupy a uniquely productive mechanistic space alongside checkpoint inhibitors. Because both platforms act through the endogenous T-cell pool, they are genuinely synergistic rather than merely additive: checkpoint inhibition sustains the T-cell function that TCEs recruit, while TCE-driven immune activation upregulates PD-L1 in ways that make checkpoint blockade more effective. This bidirectional amplification has no equivalent in CAR-T combinations, where the infused cellular product operates largely independently of the endogenous immune landscape. Manufacturing innovation is progressively reducing cost. Knob-into-hole heterodimerisation and Pichia pastoris expression systems have driven yields to approximately 2.5 g/L, with cost modelling projecting a 40% reduction in cost of goods for weekly administered Fc-silenced TCEs compared with continuous-infusion formats [53]—positioning TCEs as genuinely scalable therapies rather than interventions available only to patients in well-resourced centres.

#### 6.5.2. Where TCEs Fall Short

The most sobering limitation is durability. Despite objective response rates approaching 40% in selected populations, median progression-free survival remains modest—4.9 months for tarlatamab, with the majority of patients progressing within six to twelve months [9]. CAR-T cells can establish long-lived memory populations capable of occasional cure; TCEs cannot. The polyclonal activation that makes them broadly applicable simultaneously prevents the clonal expansion and immunological persistence that underpin durable remission [64]. Continuous or cyclical administration is therefore required to maintain target engagement, which accumulates toxicity, cost, and patient burden over time in ways that are only beginning to be characterised in longer follow-up studies.

Sustained TCE exposure also creates relentless selection pressure for antigen escape. In multiple myeloma, biallelic BCMA loss now accounts for approximately 30% of treatment failures, and soluble antigen shedding compounds the problem further—circulating BCMA acts as a pharmacological sink, sequestering therapeutic binding before it reaches tumour cells [65,93]. The very feature that makes TCEs effective—continuous immune pressure—thus undermines their own longevity in a proportion of patients that is not small.

Microenvironmental dependence is a ceiling that engineering has not yet raised. Tumours with minimal baseline CD3^+^ infiltration, dense desmoplastic stroma, or deep hypoxic niches remain largely refractory, because TCEs cannot manufacture T-cell activity from tissue that contains none [72]. CAR-T cells can be equipped with chemokine receptors—CCR2b, CXCR3—to actively traffic toward tumour deposits regardless of the local immune landscape; TCEs have no equivalent mechanism and remain dependent on conditions they cannot themselves create [64]. Finally, while ICANS rates are lower than with CAR-T therapy, the long-term neurological safety profile of continuous TCE administration—particularly for CNS-penetrant formats—is incompletely characterised, because early protocol-mandated discontinuation upon ICANS limits the follow-up data available [64]. Regulatory approval of novel multispecific constructs will require this gap to be prospectively addressed.

#### 6.5.3. Where TCEs Fit

These strengths and limitations, taken together, define a clear clinical niche. TCEs are optimally deployed where rapid disease control is needed and manufacturing time is unavailable; where lymphodepletion is contraindicated; where cellular therapy infrastructure does not exist; and where the combination of checkpoint inhibition with a second active mechanism offers an incremental survival benefit over either alone. CAR-T cells retain their advantage for younger, fit patients seeking potential cure, for tumours with homogeneous antigen expression, and for settings where the upfront toxicity and cost are justified by the prospect of deep durable remission.

The more important point, however, is that this is no longer a binary choice. The combinatorial architectures described in Section 6.4—priming, parallel delivery, sequential antigen-switching—reflect a growing clinical consensus that TCEs and cellular therapies are most powerful when deployed together, in the right order, guided by the right biomarkers. The question is no longer which platform to use. It is how to use them in sequence to give patients the best chance of a response that lasts.

### 6.6. TCEs in the Context of Standard of Care

TCEs perform best not where existing therapies work, but where they structurally fail. Across four tumour types with mature TCE evidence, a consistent pattern emerges: TCEs occupy spaces that chemotherapy, checkpoint inhibitors, and targeted agents cannot reach.

#### 6.6.1. Small-Cell Lung Cancer

Extensive-stage SCLC exemplifies oncology’s promise and frustration. Platinum-etoposide produces high initial responses, but these are transient. Adding PD-L1 inhibitors extends median survival only modestly, to ~12–13 months, because SCLC is immunologically cold—driven by neuroendocrine plasticity that suppresses antigen presentation and renders checkpoint inhibition ineffective [9].

Tarlatamab addresses this gap directly. DLL3, broadly expressed on SCLC cells but absent from normal adult tissues, provides a tumour-restricted target independent of antigen presentation or pre-existing T-cell reactivity [66]. The DeLLphi-304 trial demonstrated improved overall survival versus chemotherapy in second-line disease (median 13.6 vs. 8.3 months; HR 0.60; 95% CI 0.47–0.77; *p* < 0.001), earning FDA approval in November 2025 [53]. This represents a mechanistically distinct intervention that circumvents immunological barriers which have limited the efficacy of previous approaches. Whether tarlatamab can move earlier is now being tested in DeLLphi-305 (NCT05690945), combining TCE-driven cytotoxicity with checkpoint-sustained T-cell function.

#### 6.6.2. Metastatic Castration-Resistant Prostate Cancer

The mCRPC landscape has expanded: AR pathway inhibitors, PARP inhibitors, and lutetium-177 PSMA radioligand therapy. Yet resistance to each is inevitable: secondary AR mutations, reversion mutations, and antigen heterogeneity or internalisation for LuPSMA [81]. Patients progressing through two or three lines have exhausted most mechanisms.

TCEs bring new mechanisms. JANX007’s conditional masking means PSMA-expressing cells with LuPSMA resistance remain targetable [36]. Xaluritamig’s STEAP1 target is expressed across mCRPC subtypes, including variants refractory to hormonal strategies [44]. The XALute phase III trial compares xaluritamig against cabazitaxel in post-taxane mCRPC [100]. If positive, TCEs become a genuine alternative to cytotoxic chemotherapy where quality of life matters.

#### 6.6.3. Microsatellite Stable Colorectal Cancer

MSS CRC represents oncology’s most stubborn unmet need. Checkpoint inhibitors are inactive in MSS tumours (>85% of metastatic cases) due to low mutational burden, T-cell exclusion, and immunosuppressive myeloid landscapes. Standard care is FOLFOX or FOLFIRI with bevacizumab or cetuximab, with declining response rates and no immunotherapy option for most.

CEA-targeted TCEs engage this need directly, though cibisatamab’s 4–14% response rate reflects microenvironmental barrier depth [66]. The mechanistic understanding is clear: TGF-β-driven stromal exclusion walls T-cells away, and STING suppression prevents innate immune priming [73,82]. Combinations pairing CEA-TCB with TGF-β blockade, STING agonists, or vascular normalisation are now in evaluation [95]. MSS CRC may prove where TCE combination therapy demonstrates clearest value.

#### 6.6.4. Uveal Melanoma

Uveal melanoma carries one of the lowest mutational burdens of any cancer, is universally unresponsive to checkpoint inhibition, and historically offered response rates below 5%. The biological reason—low neoantigen burden plus MHC class I restriction—is precisely the problem TCEs were designed to solve. Tebentafusp delivered 49% mortality reduction versus investigator’s choice [85,90]. This survival benefit occurred despite only 9% radiographic response, demonstrating T-cell engagement translates into benefit through immune-mediated control not captured by conventional criteria [85,90].

Where every preceding immunotherapy failed, a TCE succeeded—not because the immune landscape was favourable, but because the TCE created one. That proof-of-concept, now extending to SCLC with tarlatamab and testing in mCRPC with xaluritamig, transforms TCEs from biologics into a platform.

#### 6.6.5. A Unifying Theme

Across these indications, the pattern is consistent. TCEs succeed where existing therapies have structurally failed—where checkpoint inhibitors cannot find T-cells, where cellular therapies cannot be delivered, where resistance has eliminated options. The challenge is deploying TCEs earlier and combining them with microenvironment-modulating strategies (Section 5.2), biomarker frameworks (Section 5.3), and cellular therapy platforms. The coming randomised trials will determine whether that ambition translates into survival outcomes patients have waited too long to see.

### 6.7. Limitations and Evidence Gaps

Several constraints temper definitive conclusions. Most solid tumour TCE data derive from single-arm phase I/II studies with median enrolment of 40–100 patients and follow-up under 12 months, limiting durability assessment [74]. Antigen density thresholds were predominantly derived retrospectively; prospective validation in registration trials remains incomplete [70,94]. The pharmacokinetics of masked and multispecific constructs are incompletely characterised, with optimal step-up dosing schedules unexamined in randomised comparisons. Combination strategy evidence, while mechanistically compelling, derives largely from single-arm expansion cohorts without placebo controls, introducing potential for selection bias [82,97]. Long-term neurotoxicity surveillance is constrained by early protocol-mandated discontinuation upon ICANS [24]. Health economic assessments rely on modelling (incremental cost-effectiveness ratios ≤ US$150,000/QALY) rather than mature phase III data [76].

### 6.8. Regulatory, Commercial, and Access Considerations

Regulatory frameworks have evolved pragmatically. Step-up dosing protocols constitute a cornerstone of cytokine release syndrome mitigation, established through FDA collaboration on tarlatamab [100]. Companion diagnostic development reflects flexibility: despite FDA approval of the VENTANA DLL3 (SP347) assay, tarlatamab’s label permits treatment regardless of baseline expression, acknowledging efficacy across antigen levels [63,98]. For orphan indications, evidentiary standards remain stringent. Tebentafusp’s approval required overall survival as primary endpoint—quality-of-life data were supplementary evidence—a precedent underscoring that survival advantage remains decisive even in ultra-rare diseases [85,90]. Manufacturing innovations are reducing cost of goods. Knob-into-hole engineering and *Pichia pastoris* expression systems have driven yields to 2.5 g/L [11,53]. Cost modelling predicts 40% reductions for weekly administered Fc-silenced TCEs versus continuous-infusion formats [11,53].

### 6.9. Outlook: Toward 2030

The coming decade will determine whether TCEs become foundational solid tumour therapies. Randomised trials comparing TCE plus tumour microenvironment modulators against monotherapy will read out by 2027–2028, clarifying incremental value [82]. The DeLLphi-305 trial (tarlatamab plus PD-L1 maintenance in first-line ES-SCLC) and XALute trial (xaluritamig versus cabazitaxel in post-taxane prostate cancer) represent critical efficacy benchmarks [44,55,101]. Earlier disease settings offer substantial opportunity. The first adjuvant TCE trial—masked anti-PSMA construct versus observation in high-risk post-prostatectomy prostate cancer—opens in 2026 [36]. Paediatric precision-oncology initiatives will evaluate B7-H3-directed constructs in neuroblastoma and Ewing sarcoma [60,62]. Convergence with cellular immunotherapy platforms—allogeneic CAR-T cells combined with TCEs—enters early clinical development [64,65]. By 2030, biomarker-stratified, sequentially activated regimens will likely become standard: TCEs primed by brief microenvironment modulation, dosed via step-up protocols, guided by real-time immune profiling. With tarlatamab established in second-line SCLC and xaluritamig phase III readouts imminent, TCEs are positioned to become standard-of-care options in biomarker-defined solid tumours. Realising these potential demands sustained cross-disciplinary collaboration—and unwavering commitment to equitable global access.

TCEs have crossed the threshold from proof-of-concept to platform therapy in solid tumours. The coming decade will determine the breadth, durability, and ultimate clinical role of that platform.

## 7. Conclusions

The therapeutic landscape for solid tumours is undergoing a profound transformation. Next-generation CD3-based T-cell engagers have matured from experimental haematology agents into sophisticated, biomarker-directed immunotherapies that actively engage with the tumour microenvironment. Conditional activation strategies—ranging from protease-masked prodrugs to hypoxia-gated formats—have already reshaped the safety and efficacy profiles of these agents, delivering objective response rates approaching 40–50% in molecularly selected populations such as DLL3-positive small-cell lung cancer, while maintaining grade ≥ 3 cytokine release syndrome rates below 3% through optimised dosing. Following the pathbreaking precedent of tebentafusp in uveal melanoma, these advances are now translating into tangible survival benefits in phase III settings, with traditional regulatory approval secured for tarlatamab and pivotal trials advancing for prostate-selective agents. What emerges from this trajectory is something more than incremental progress. Molecular engineering, biomarker-driven selection, and tumour microenvironment modulation have stopped behaving as parallel developments and started converging into a unified framework—one that is actively reshaping TCEs from narrowly applicable experimental agents into genuine platform therapies capable of reaching patients across multiple solid tumour types. Yet the biology of resistance remains formidable. Antigen escape, physical exclusion by desmoplastic stroma, and T-cell exhaustion continue to limit durability in early-phase studies. What is striking about the current moment is that none of these barriers remains without a rational engineering response. Antigen loss meets trispecific constructs, stromal exclusion meets conditional masking and vascular normalisation, and T-cell exhaustion meets IL-15 fusions and 4-1BB costimulation. For the first time, resistance biology is functioning less as an obstacle and more as a design specification for next-generation TCE platforms. Crucially, the field now possesses rational countermeasures for each axis: multi-target and trispecific constructs to outmanoeuvre antigen loss; innate immune priming through oncolytic viruses, STING agonists, or CD40-targeting adjuvants to remodel the extracellular matrix; and orthogonal payloads such as IL-15 super-agonists or checkpoint-inhibitory scFvs to rejuvenate exhausted effector populations. This modular engineering paradigm positions TCEs not as static drugs, but as adaptable platforms capable of iterative refinement across diverse oncologic contexts.

Looking toward the horizon, 2030 promises a fundamental realignment of the treatment paradigm. Phase III readouts for agents like xaluritamig, alongside emerging adjuvant and paediatric programmes, will determine whether TCEs anchor second-line therapy in biomarker-defined populations and extend into curative-intent settings. The vision—biomarker-stratified, sequentially activated regimens combining TCEs with short-course tumour microenvironment modulation and guided by real-time immune profiling—is fast becoming tangible reality. Delivering on this promise demands more than scientific ingenuity; it requires sustained cross-disciplinary collaboration among molecular engineers, translational immunologists, trialists, and regulatory scientists. Immediate priorities include prospective validation of composite biomarker algorithms, harmonisation of complementary diagnostics such as the VENTANA DLL3 assay, rigorous long-term safety surveillance, and—critically—ensuring equitable global access. For populations in low- and middle-income countries, where diagnostic infrastructure and cost remain prohibitive barriers, the challenge is as much about delivery as discovery. With continued innovation grounded in these collaborative principles, the coming generation of T-cell engagers holds genuine potential to deliver durable clinical benefit and, ultimately, transformative cancer control for solid tumours.

## Figures and Tables

**Table 1:** Clinically advanced CD3-based T-cell engagers in solid tumours.

Agent	Molecular Architecture	Tumour-Associated Antigen	Lead Indication	Representative Clinical Activity *	Development Status
Tarlatamab	Half-life-extended bispecific antibody	DLL3	Extensive-stage SCLC	ORR ~ 40%; mOS ~ 13.6 months	FDA approved
Xaluritamig (AMG 509)	IgG-like bispecific antibody	STEAP1	Metastatic CRPC	ORR ~ 41–43%; high PSA response rates	Phase II
JANX007	Conditionally activated (masked) bispecific	PSMA	Metastatic CRPC	PSA50 ~ 70%; confirmed radiographic responses	Phase I
Cibisatamab	IgG-based bispecific antibody	CEA	MSS colorectal cancer	ORR 4–14% across dose levels	Phase I/II

* Data from ongoing or preliminary clinical studies; results subject to change as trials progress.

**Table 2:** Selected TME-targeted combination strategies with CD3 TCEs.

Modulatory Strategy	Primary Biological Effect	Key Supporting Evidence	Translational Rationale	References
γ-Secretase inhibition	Increased surface antigen density	2–3-fold DLL3 upregulation	Improves target engagement and synapse stabilisation	[65,66]
STING pathway activation	Innate immune priming and DC activation	Increased intratumoural CD8^+^ T-cell infiltration	Converts immune-cold tumours to permissive states	
Oncolytic virotherapy	Stromal remodelling and antigen release	Enhanced immune infiltration in fibrotic tumours	Facilitates TCE penetration	[67]
Anti-angiogenic therapy	Vascular normalisation	Improved immune trafficking and drug delivery	Synergizes with T-cell-dependent therapies	[71,72]

**Table 3:** Biomarkers for patient selection and pharmacodynamic monitoring of T-cell engagers.

Biomarker Category	Measurement Modality	Observed/Proposed Threshold	Clinical Utility	Reference
Target antigen expression	Quantitative IHC/Flow cytometry	≥10,000 copies per cell or H-score ≥ 150	Predicts likelihood of objective response	[72]
Baseline T-cell infiltration	CD3^+^ cells per mm^2^	≥250 cells/mm^2^	Associated with 2-fold higher ORR	[73]
Early immune activation	Soluble IL-2 receptor α	Transient post-dose elevation (cycle 1, day 2)	Pharmacodynamic marker and grade ≥ 2 CRS risk predictor	[71]
Myeloid cell burden	CD68^+^ macrophage density	≥30% of immune infiltrate	Negative predictor of efficacy (8% vs. 31% ORR)	[85]

**Table 4:** Design principles governing efficacy and durability of CD3-based T-cell engagers.

Biological Dimension	Limiting Factor in Solid Tumours	Supporting Clinical/Translational Evidence	Consequence for Clinical Outcomes	Rational Design Strategy
Target antigen biology	Low, heterogeneous, or adaptive antigen expression	Antigen downregulation in 28–60% of progressing tumours	Incomplete synapse formation; early relapse	Multispecific targeting; pharmacologic antigen upregulation
Tumour microenvironment architecture	Stromal density and immune exclusion	Reduced activity in desmoplastic tumours	Limited T-cell and drug penetration	Sequential TME priming strategies
Myeloid-driven suppression	Expansion of MDSCs and TAMs	High CD68^+^/CD163^+^ infiltrates correlate with resistance	Attenuated cytotoxic function	Myeloid-modulating combinations
T-cell functional state	Exhaustion following sustained CD3 signalling	PD-1, LAG-3, TOX induction despite initial response	Short response durability	Costimulatory enhancement; cytokine support
Systemic immune activation	Rapid cytokine release on first exposure	Early soluble IL-2Rα elevation predicts CRS	Dose-limiting toxicity	Step-up dosing; conditional activation

**Table 5:** Phase III and late-stage pipeline of next-generation TCEs and combinations.

Agent/Strategy	Molecular Target/Design	Disease Setting	Clinical Stage	Key Clinical Signals	Interpretive Commentary	Ref.
Tarlatamab	DLL3 × CD3	2nd-line ES-SCLC	Phase III (DeLLphi-304)	OS 13.6 vs. 8.3 mo; HR 0.60 (95% CI 0.47–0.77); *p* < 0.001	First T-cell engager to demonstrate a statistically significant OS benefit in a solid tumour; FDA approved in 2025	[53]
Tarlatamab + Atezolizumab	DLL3 × CD3 + PD-L1 blockade	1st-line ES-SCLC maintenance	Phase Ib/II (DeLLphi-303); Phase III ongoing (DeLLphi-305)	Early survival trend; mPFS 5.6 mo; DCR 62.5%	Checkpoint inhibition may sustain T-cell function during prolonged engager therapy	[52]
Xaluritamig (AMG 509)	STEAP1 × CD3	Post-taxane mCRPC	Phase III (XALute; NCT06691984)	rPFS/OS pending	First registrational TCE trial in prostate cancer versus standard chemotherapy	[44]
JANX007	PSMA × CD3 (conditionally masked)	Metastatic CRPC	Phase I/II (ENGAGER-PSMA-01)	PSA50 ~ 70%; radiographic responses; low-grade CRS	Conditional activation improves therapeutic index in solid tumours	[36]
Cibisatamab + Atezolizumab	CEA × CD3 + PD-L1 blockade	MSS colorectal cancer	Phase I/II (CO40939; NCT03866239)	ORR ~ 14% (flat-dose cohort)	Modest activity supports checkpoint–TCE combination strategy	[11]
RO7122290 + Cibisatamab	FAP-4-1BBL costimulation + CEA × CD3	MSS colorectal cancer	Phase I/II (NCT04826003)	Safety; immune infiltration	Stromal-targeted costimulation may enhance intratumoral T-cell activation	[102]

**Table 6:** Scheduling architectures for TCE–cellular therapy combinations in solid tumours.

Combination Model	Cellular Partner	Mechanistic Basis	Optimal Clinical Scenario	Key Practical Consideration	References
Prime-then-Engage	CAR-T	Short TCE course expands tumour-infiltrating T-cells, upregulates inflammatory chemokines, and reduces suppressive myeloid burden before adoptive cell infusion	Cold tumours; baseline CD3^+^ infiltration < 250 cells/mm^2^; desmoplastic stroma restricting CAR-T trafficking	Optimal TCE duration and washout interval undefined; risk of depleting TCF1^+^ progenitor T-cell pool prior to infusion	[64,82]
Parallel Co-Administration	CAR-T or CAR-NK	TCE redirects endogenous polyclonal T-cells; cellular product delivers independent cytotoxicity via a separate antigen arm	Antigen-heterogeneous tumours with ≥2 co-expressed targetable epitopes; post-first-line relapsed or refractory settings	Overlapping cytokine release requires enhanced monitoring; CAR-NK orthogonality (no CD3ε) substantially reduces CRS amplification risk	[64]
Sequential Antigen-Switching	CAR-T → TCE	CAR-T (antigen A) eliminates primary clone; TCE (antigen B) re-engages antigen-loss escape variants or biallelic deletion subclones after CAR-T failure	Post–CAR-T progression with confirmed antigen loss or biallelic deletion (e.g., BCMA or GPRC5D in multiple myeloma)	Requires prospective dual-antigen tumour profiling at baseline; optimal switch timing governed by real-time pharmacodynamic monitoring	[64,93]
Pharmacological Priming + Cellular Therapy	CAR-T or CAR-NK	γ-Secretase inhibition or analogous agents upregulate surface antigen density before TCE or cellular therapy, lowering the recognition threshold	Low or heterogeneous surface antigen expression limiting TCE synapse formation or CAR-T engagement efficiency	33-fold BCMA upregulation established in myeloma [90]; translational evaluation of DLL3/STEAP1 upregulation in solid tumours required	[65,93]

CAR-T, chimeric antigen receptor T-cell; CAR-NK, chimeric antigen receptor natural killer cell; TCE, T-cell engager; CRS, cytokine release syndrome; TCF1^+^, T-cell factor 1-positive progenitor-exhausted T-cells; BCMA, B-cell maturation antigen; GPRC5D, G protein-coupled receptor class C group 5 member D; CD3ε, CD3 epsilon chain.

## Data Availability

The data generated during this study are included in this article.
